# Zika Virus Neuropathogenesis: The Different Brain Cells, Host Factors and Mechanisms Involved

**DOI:** 10.3389/fimmu.2022.773191

**Published:** 2022-03-16

**Authors:** Thamil Vaani Komarasamy, Nur Amelia Azreen Adnan, William James, Vinod R. M. T. Balasubramaniam

**Affiliations:** ^1^ Infection and Immunity Research Strength, Jeffrey Cheah School of Medicine and Health Sciences, Monash University Malaysia, Bandar Sunway, Malaysia; ^2^ Sir William Dunn School of Pathology, University of Oxford, Oxford, United Kingdom

**Keywords:** Zika virus, host factors, microcephaly, Guillain-Barre syndrome, immune response, neuroinflammation, mitochondrial damage, animal models

## Abstract

Zika virus (ZIKV), despite being discovered six decades earlier, became a major health concern only after an epidemic in French Polynesia and an increase in the number of microcephaly cases in Brazil. Substantial evidence has been found to support the link between ZIKV and neurological complications in infants. The virus targets various cells in the brain, including radial glial cells, neural progenitor cells (NPCs), astrocytes, microglial and glioblastoma stem cells. It affects the brain cells by exploiting different mechanisms, mainly through apoptosis and cell cycle dysregulation. The modulation of host immune response and the inflammatory process has also been demonstrated to play a critical role in ZIKV induced neurological complications. In addition to that, different ZIKV strains have exhibited specific neurotropism and unique molecular mechanisms. This review provides a comprehensive and up-to-date overview of ZIKV-induced neuroimmunopathogenesis by dissecting its main target cells in the brain, and the underlying cellular and molecular mechanisms. We highlighted the roles of the different ZIKV host factors and how they exploit specific host factors through various mechanisms. Overall, it covers key components for understanding the crosstalk between ZIKV and the brain.

## Introduction

**Figure 1:**
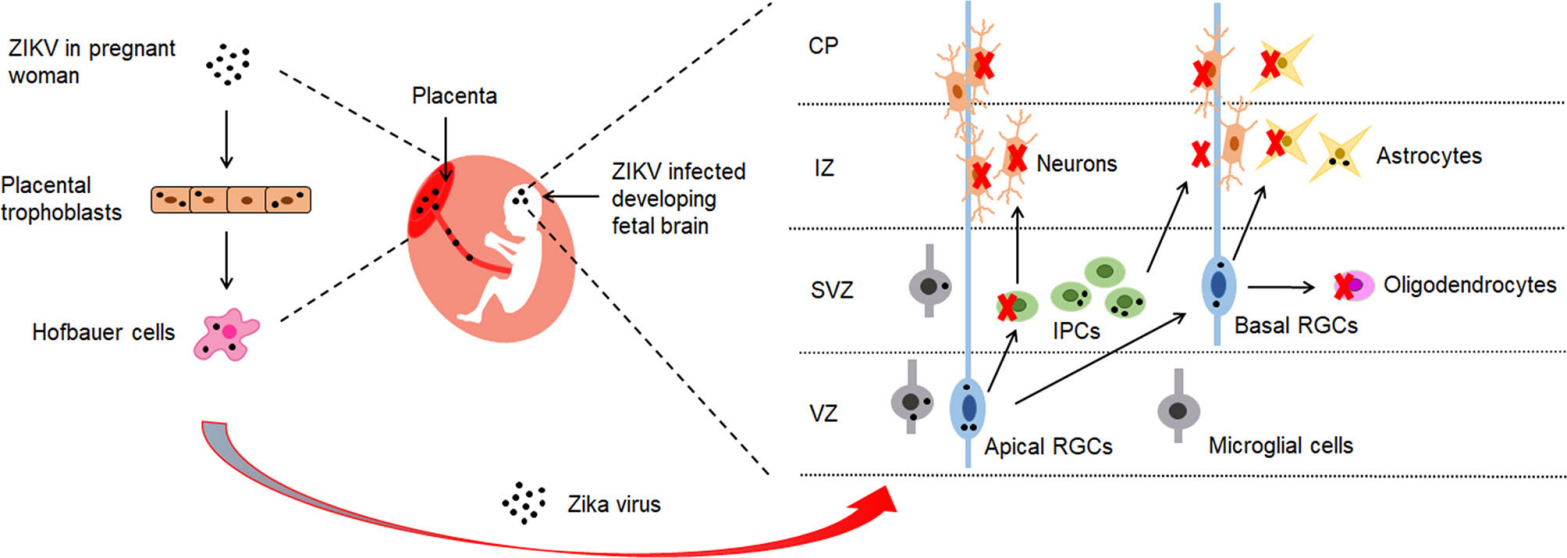
The brain cells targeted by ZIKV. ZIKV has shown to possess the ability to cross the placental barrier of infected pregnant women (8). It reaches fetal brains through placenta by infecting placental trophoblasts and Hofbauer cells (7, 9). In the developing fetal brain, ZIKV has demonstrated to mainly target radial glial cells (RGS) in the ventricular zone (VZ) and intermediate progenitor cells (IPCs) in the sub-ventricular zone (SVZ) (8, 10). During brain development, cortical stem cells give rise to radial glial cells (RGCs) which proliferate into progenitor cells that make up the brain and central nervous system. RGCs generate IPCs which divide and generate the majority of neurons in the brain. The neurons migrate through intermediate zone (IZ) to the developing cortical plate (CP) (11, 12). ZIKV infection in the fetal brain has resulted in depletion of IPCs and differentiating neurons, and has caused reduction of thickness of VZ, SVZ and CP layers (13). Numerous studies provide evidence that ZIKV has shown to induce apoptosis, cell cycle dysregulation and immune response in neuronal cells, which leads to impaired neurogenesis and microcephaly.

Zika virus (ZIKV) infection has been associated with adverse pregnancy and birth outcomes with numerous neurological complications (1–4). Microcephaly is the most obvious symptom associated with congenital Zika syndrome (CZS). It is characterized by reduced brain size and volume, abnormal development of the neurons and reduced number of neurons in the grey matter (5, 6). To reach the fetal brain, ZIKV needs to cross the placental and the blood-brain barrier (BBB), which are responsible for protecting fetal brain development from pathogens during pregnancy. Chronic placentitis has been observed in ZIKV-infected pregnant women (7). Evidence suggests that ZIKV crosses the placenta to reach the fetus by directly infecting placental cells and disrupting the placental barrier (**Figure 1**). It is hypothesized that ZIKV might reach the fetal vessels by using the migratory ability of Hofbauer cells (**Figure 1**). Placental trophoblasts have also been shown to be another target cell for ZIKV infection (9, 14) (**Figure 1**).

Recent studies have demonstrated that ZIKV crosses the BBB by infecting the brain endothelial cells and altering the tight junction proteins (15–17). However, the mechanism of ZIKV crossing the BBB to the central nervous system (CNS) is still unclear. Initial studies suggested that ZIKV crosses the BBB through the basolateral release or transcytosis pathway without BBB disruption. However, it was found that persistent ZIKV infection and inflammatory response may lead to disruption of the BBB at later time points (15, 17). Another study showed that the disruption of the BBB permeability by ZIKV could be strain dependent (16). Alternatively, a recent study has demonstrated that ZIKV might exploit the blood-cerebrospinal fluid (B-CSF) by infecting pericytes in the choroid plexus (18). Once the virus reaches the developing fetal brain, it has been shown to mainly infect cells in the ventricular zone (VZ) and subventricular zone (SVZ) (8) (**Figure 1**). The VZ consists of neuroepithelial cells (NECs) and radial glial cells (RGCs), while the SVZ contains intermediate progenitor cells (IPCs) (19). RGCs give rise to neurons, astrocytes, ependymal cells and oligodendrocytes. IPCs generate neurons or glial cells, including oligodendrocytes and astrocytes (20).

To further support the association between ZIKV and the brain, ZIKV-host protein-protein interaction (PPI) studies have identified cellular proteins involved in neurogenesis, embryonic and central nervous system (CNS) development as well as neurological disorders. In light of this, Scaturro et al. identified key ZIKV cellular targets such as LARP7, LYAR, NGDN, CLN6, BSG, CEND1, RBFOX2, CHP1 and TMEM41b (21). These identified proteins play important roles in neuronal development (22), embryonic growth and development (23–26), nervous system development (27) and have been implicated in neurogenerative (28) and developmental disorders (29). ZIKV protein was also found to interact with ANKLE2, a gene associated with brain development in both drosophila and humans (30, 31). The key host factors that mediate ZIKV neuropathogenesis discussed in this review are listed in **Table 1**.

**Table 1:** Host factors that mediate ZIKV neuropathogenesis.

ZIKV protein	Host protein	Function
Capsid	LARP7	Knockdown of LARP7 showed reduction of neuronal ribosome content as well as inhibition of protein synthesis in the hippocampal neurons; mutations in LARP7 are linked to microcephaly (29).
	LYAR	A nucleolar protein that plays a role in cell growth is highly expressed during embryonic development and in undifferentiated human embryonic stem cells (ESCs) (23, 24).
	NGDN	An important translational regulatory protein during the development of the vertebrate nervous system (27).
NS2A	Adherens junctions (AJs)	Regulates signaling pathways critical for neural development and its disruption is associated with architectural disorganization of the developing cortex (32).
NS3	CEP192	A major regulator of centrosome biogenesis and spindle organization (33); associated with microcephaly (34).
	CEP85	A regulator of centriole duplication (35).
	OFD1	A centriolar satellite protein and regulator. of centriole architecture; critical for forebrain development (36).
NS4A	ANKLE2	Associated with brain development; its mutations causes microcephaly (18); ZIKV-NS4A resulted in reduction of brain size, affected neuroblast division and brain development in Drosophila by targeting the ANKLE2 pathway (8, 19).
NS4B	BSG	Critical in fetal development and retinal function (37, 38); its knockdown of BSG inhibited ZIKV replication (21).
	CLN6	Contributes to lysosomal function as well as the viability of neurones (22); mutations in CLN6 have shown to cause neurodegenerative disease (28); CLN6 is also associated with mTOR and TELO2 regulators of signaling pathways that are known to be disrupted by ZIKV (21).
	CENDI	Functions as an inducer of neuronal differentiation in neuronal precursor cells (39); knockdown of CENDI inhibited ZIKV replication (21).
	RBFOX2	An important role in splicing regulation during embryonic growth and development; deficiency of the protein has shown to cause reduced cerebellar size (26).
	TMEM41b	Associated with motor system dysfunction in neurodegenerative disorder and has been shown to be essential in mouse embryonic development (25); its knockdown inhibited ZIKV replication (21).
NS5	STAT2	Involved in antiviral immunity and regulation of IFN-I signaling (40); ZIKV inhibits IFN signaling through STAT2 degradation (41).
	CDK5RAP2	Plays a critical role in cell cycle; loss of CDK5RAP2 function associated with reduced numbers of neural progenitor cells (NPCs); mutations in CDK5RAP2 are linked to primary microcephaly (42, 43); mutations in CDK5RAP2 were identified in case of vertically transmitted ZIKV infection with congenital syndrome (44).
	TBK1	Highly expressed in NES cells and RGCs/IPCs in the developing neocortex; essential for both innate antiviral immune signaling and for cell proliferation; ZIKV infection caused relocation and sequestration of pTBK1 from centrosomes to mitochondria (45).
NS4A/NS5	Doublecortin (DCX)	A microtubule-associated protein; involved in neurogenesis; downregulated at both mRNA and protein levels during ZIKV infection in NPCs and fetal mouse brains (46).
Unknown	PTPRZ1	Expressed mainly in the CNS during development; increased levels in ZIKV-infected brains (1).
	MFN2	Highly expressed in the brain (47); essential in embryonic development (48); maintains the integrity of mitochondrial morphology and mediates mitochondria fusion; ZIKV disrupts mitochondrial dynamics by targeting MFN2 (49).

This review provides a comprehensive and up-to-date overview of ZIKV-induced neuropathogenesis by dissecting the underlying cellular and molecular mechanisms. We explored in detail how ZIKV infection and the mechanisms involved are dependent on the types of strains, cells and infection rate. Here we provide the most updated evaluation of recently identified key host proteins responsible for the neuropathogenesis of ZIKV (**Table 1**). We highlighted the roles played by the different ZIKV and how they exploit specific host proteins through various mechanisms. Overall, it covers some of the key components to understand the crosstalk between ZIKV and the brain.

## ZIKV Entry Receptors

**Figure 2:**
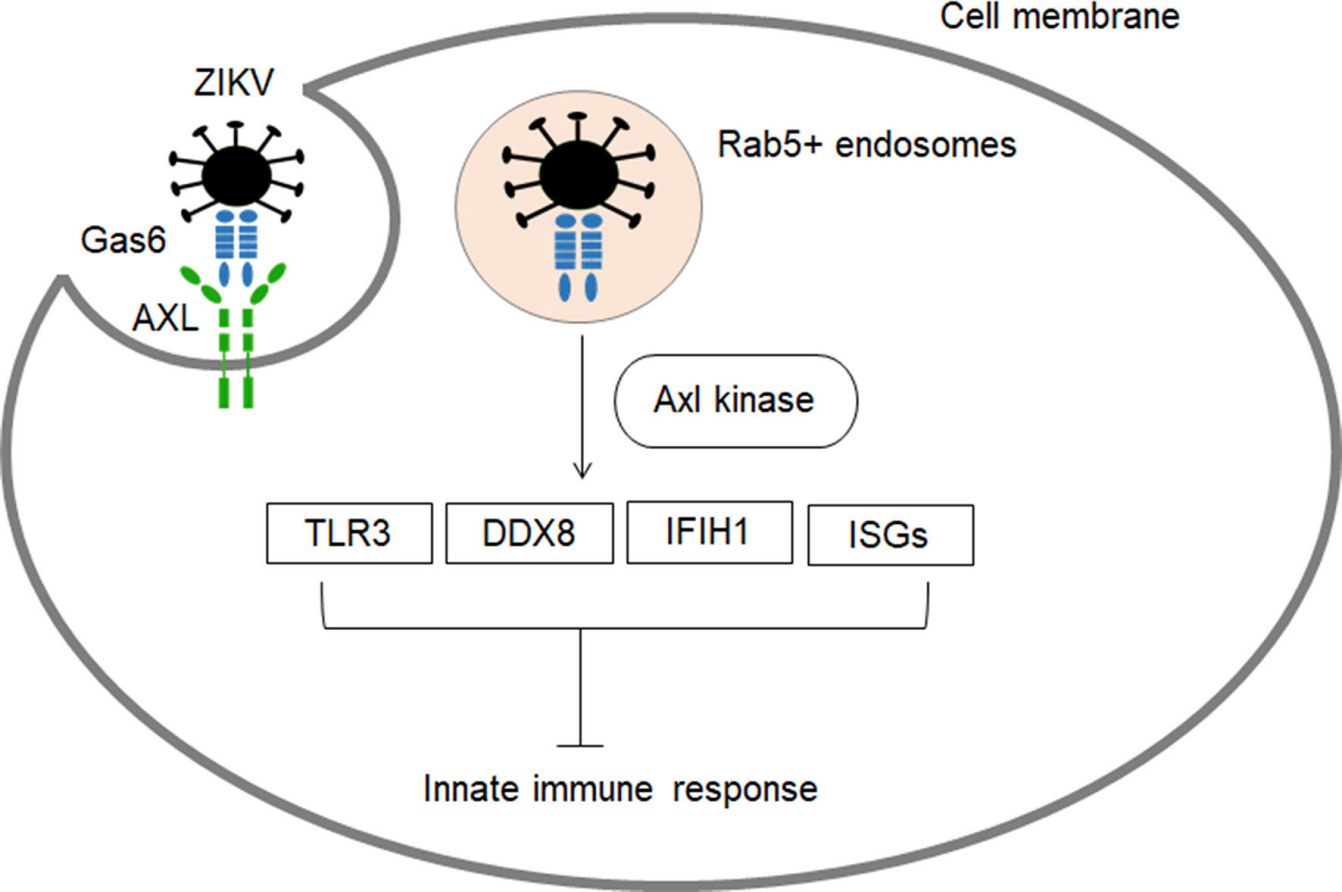
The role of AXL receptor in ZIKV entry and subsequent inhibition of innate immune response. AXL are highly expressed in developing human brain cells (radial glia, astrocytes, endothelial and microglia), making these cell types particularly vulnerable to ZIKV infection (8, 52). ZIKV binds to Gas6 and uses Axl to gain entry into cells (53). Subsequently, the virus dampens innate immunity through activation of Axl kinase which induces the transcription of TLR3, DDX58, and IFIH1 as well as several interferon-stimulated ISGs (53, 54).

AXL, a receptor tyrosine kinase that has been implicated in multiple cellular responses (50) and regulation of inflammatory responses (51), plays a critical role in ZIKV entry (**Figure 2**). Firstly, the virus is recruited to the AXL receptor through TAM ligand growth arrest specific 6 (Gas6), which results in internalization of ZIKV through clathrin-mediated endocytosis (53). Then, the vesicles containing ZIKV are translocated to Rab5+ endosomes. The ZIKV/Gas6 complex activates AXL kinase activity which induces the transcription of TOLL-like receptor 3 (TLR3), DExD/H-Box helicase 58 (DDX58), and interferon induced with helicase C domain 1 (IFIH1) as well as several interferon-stimulated genes (ISGs), leading to suppression of the innate immune response and subsequent productive infection (**Figure 2**) (53, 54). The developing human brain cells such as radial glia, astrocytes, endothelial and microglia overexpress AXL protein, and they are especially vulnerable to ZIKV infection (52). Blocking of AXL has been shown to inhibit ZIKV infection in glial cells (53, 55). In astrocytes, pre-treatment of the cells with antibodies targeting AXL managed to reduce ZIKV infection (56). In addition, murine microglial cells with weak expression of AXL were shown to be resistant to ZIKV (53). However, evidence supports that AXL is not indispensable for ZIKV entry, and its role in ZIKV infection could be cell type-specific. AXL knockout mice showed similar levels of ZIKV RNA as compared to wild-type mice (57). It has been proven that AXL is not required for ZIKV infection in neural progenitor cells (NPCs) (53, 58). Hence, there could be additional receptors that aid ZIKV entry into specific cells.

A recent study identified the role of Neural Cell Adhesion Molecule (NCAM1) in ZIKV entry. The study demonstrated that inhibition of the NCAM1 receptor significantly reduced ZIKV attachment and entry while its overexpression enhanced ZIKV attachment and internalization (59). However, the role of NCAM1 in ZIKV entry should be further investigated in different cell types. Pujhari et al. demonstrated the role of Hsp70 in ZIKV binding and entry into mammalian cells. Blocking of cell surface-localized HSP70 reduced ZIKV infection and production of infectious virus particles (60). HSP70 is also shown to mediate post-entry steps, including ZIKV replication, capsid assembly and egress in various cells such as human neural stem cells (hNSCs) and placental trophoblast cells (60, 61). A study by Khongwichit et al. showed interaction between GRP78 and ZIKV E protein and found that antibodies blocking the N-terminus of GRP78 as well knockdown by siRNA inhibited ZIKV entry into host cells (62). In another study, the cell surface α2,3-linked sialic acid was found to mediate ZIKV internalization. Removal of cell surface sialic acid using neuraminidase inhibited ZIKV infection in Vero cells and human iPSC-derived NPCs, suggesting its role in facilitating ZIKV internalization. In addition, α2,3-linked sialic acid-knockout significantly reduced ZIKV infection. The study also showed that sialic acid does not directly interact with ZIKV and it is not involved in ZIKV attachment (63). **Table 2** provides the host factors that facilitate ZIKV entry into the host cells.

**Table 2:** Host factors that facilitate ZIKV entry into different host cells.

ZIKV protein	Targeted host protein	Function
ENV	AXL	Highly expressed in human radial glial cells, astrocytes, microglial and endothelial cells in the developing brain; promotes ZIKV entry and modulates innate immune responses (52, 53).
	NCAM1	Shapes the neuronal network during development; involved in synaptic plasticity and cognitive functions (64, 65); facilitates ZIKV attachment and entry into host cells (59).
	Integrin αvβ5	Internalization factor for ZIKV in human neural stem cells (hNSCs) and glioblastoma stem cells (GSCs) (66).
	GRP78	Endoplasmic reticulum (ER) chaperone protein GRP78; facilitates proper folding of nascent proteins; involved in ER stress by mediating the unfolded protein response (UPR) pathway (67); mediates ZIKV entry into host cells (62).
Capsid	Hsp70	Plays an essential role in protein translation, folding, intracellular trafficking, and degradation; involved in signal transduction, apoptosis, cell cycle regulation and innate immunity (68); implicated in the replication of various viruses (69); facilitates ZIKV binding and entry into mammalian cells as well as post-entry processes including replication and egress (60, 61).
Unknown	α2,3-linked sialic acid	Attachment or entry receptor for various viruses; mediates ZIKV internalization (63).

## Strains Linked to Microcephaly

Different flaviviruses and almost all strains of ZIKV have demonstrated the ability to infect NPCs and induce apoptosis in cell models. However, only certain strains of ZIKV have been shown to induce neurological disorders, including microcephaly in animal models (70, 71) (**Table 3**). Animal models better represent actual brain tissue and could mimic at least some clinical features of ZIKV infection in pregnant women, as well as newborns. Therefore, in addition to cell models it is crucial to study ZIKV infection in animal models to gain a deeper understanding of the virus-induced neuropathogenesis. A number of studies have been conducted using various animal models to evaluate the neuropathogenesis of ZIKV. **Table 4** provides the list of animal models mentioned in this review to provide evidence of ZIKV-induced neuropathogenesis and to understand the various mechanisms involved. All the ZIKV strains listed in **Table 3** consist of S139N substitution in the pre-membrane (prM) region, which is associated with increased infectivity and microcephaly (75). A sequence alignment of the prM region of all five strains exhibited 100% identity. A single mutation in the prM region of a contemporary ZIKV strain (VEN/2016) resulted in increased ZIKV infectivity in NPCs and more severe microcephaly, as well as higher mortality in the mouse model compared to an ancestral Asian strain (Cam/2010) without the S139 substitution. In addition, a single reverse mutation in VEN/2016 resulted in a significant reduction in mortality in neonatal mice (75).

**Table 3:** ZIKV strains that causes microcephaly and other neurological complications in animal models.

ZIKV strains	Signs of microcephaly and other neurological complications	Reference
Brazil-ZKV2015	▪ Pups born to the infected pregnant mice (SJL mice) displayed intra-uterine growth restriction (IUGR) and ocular abnormalities. ▪ Infected mice brains exhibited cortical malformations with reduced cell number and cortical thickness. ▪ Resulted in a reduction of proliferative zones and disrupted cortical layers in human brain organoids.	(72)
MEX1-44	▪ Replicated effectively in mouse brains (C57BL/6J from developmental through postnatal stages resulting in smaller body and brain size. ▪ Induced growth restriction of brain and other organs (hearts, lungs, livers and kidneys). ▪ Caused reduction in cortical radial thickness in infected brains as well as reduction in total number of neurons.	(73)
SZ01	▪ Replicated efficiently in embryonic mouse brains (ICR mice) and resulted in smaller sized brains with thinning of cortical layers	(74)
VEN/2016	▪ Displayed 100% mortality in neonatal mice (BALB/c mice) with neurological indications including inactivity, motor weakness, and bilateral hind limb paralysis. ▪ Caused microcephaly with cortical thinning in embryonic littermate brains	(75)
ZIKV_SMGC-1	▪ Reduced birth rate of the infected neonatal mice (C57BL/6) to 71.9%. ▪ Viral RNA was detected in kidneys, eyes, and spinal cords of some offspring at postnatal day 0. ▪ 74.3% of the infected offspring mice died before postnatal day 28. ▪ Smaller brain size was observed in infected infant mice at postnatal day 14. ▪ 29.1% of cells in the infant cortex were positive for ZIKV and cspase-3. ▪ Infected infant mice displayed smaller eyeballs and thinner optic nerves with visual deficiencies. ▪ Caused hind limb paralysis in offspring mice.	(71)

**Table 4:** Mouse models for ZIKV neuropathogenesis.

Model strain	Age	Virus inoculation route/dosage	ZIKV strains	ZIKV detected	Pathologies	Ref.
Ifnar1-/- or Irf3−/−Irf5−/− Irf7−/− triple KO	5–6 weeks	s.c. (footpad) or i.p. or i.v./1 × 10^3^ FFU	H/PF/2013, MR 766	Serum, spleen, brain, spinal cord, and testes	Hindlimb paralysis, hindlimb weakness, death	(76)
C57BL/6 treated with IFNAR1 antibody	4–5 weeks	i.p. or s.c./1 × 10^3^–10^6^ PFU	strain DAK AR D 41525	Spleen, liver, kidney, heart, brain, spinal cord	Neuronal death, astrogliosis, microgliosis, scattered necrotic cellular debris, inflammatory cell infiltrates	(77)
Pregnant CD1	E10	i.u. 1x10^6^ TCID_50_ units/100uL	FSS13025, Paraiba 2015 (ZIKV^BR^), PRVABC59	Trophoblast and endothelial cells in the placenta, and endothelial, microglial and NPCs in the fetal brain	Placental inflammation and dysfunction, reduced fetal viability, neuroinflammation and cortical thinning in neonatal brains	(78)
Pregnant SJL	E10-13	i.v./2 × 10^2^, 8 × 10^9^, or 2 × 10^11^ PFU	Paraiba 2015 (ZIKV^BR^)	Fetal brain, kidney, liver, spleen	Apoptosis in neural tissue, IUGR, cortical malformations similar to microcephaly	(72)
Pregnant C57	E13.5	i.p./9 × 10^4^ PFU	SZ01	Serum, placenta, fetal brain	Reduction of the cortical NPCs in the fetal mice.	(13)
					Reduced cavity of lateral ventricles and surface areas of the cortex.	
Pregnant C57BL/6J or 129S1/SvImJ	E14.5	i.c./1.7 × 10^3^ TCID50	MEX1-44	N/A	Postnatal growth restriction and microcephaly with neuronal loss, cell cycle arrest and apoptosis of NPCs, dysregulation of genes associated with immune responses in the brains, abnormal vascular development, BBB leakage, microglial activation, astrogliosis	(73)
ICR	E13.5	Lateral ventricle (injection of fetus)/650 PFU	SZ01	Fetal brain	apoptosis and cell-cycle arrest of NPCs, deregulation of associated with immune response, apoptosis pathways and microcephaly in the brains, Smaller brain size with enlarged ventricles and a thinner CP and VZ/SVZ	(74)
Ifnar1^+/−^ (IFNAR1-/- × C57BL/6)	E6.5 or E7.5	s.c. (footpad)/1 × 10^3^ PFU	H/PF/2013	Placenta, fetal head and body, maternal serum, spleen and brain	placental and fetal brain apoptosis, fetal demise, IUGR	(79)
Ifnar1^+/−^ (C57BL/6 treated with anti-Ifnar antibody [MAR1-5A3])	E6.5 or E7.5	s.c. (footpad)/1 × 10^3^ PFU	H/PF/2013	Placenta, fetal head and body, maternal serum, spleen and brain	IUGR	(79)

Ifnar, interferon-α/β receptor; IFR, interferon regulatory factor; NPCs, neural progenitor cells; IUGR, intra-uterine growth restriction; TCID50, 50% median tissue culture infectious dose; PFU, plaque forming units; CP, cortical plate; VZ, ventricular zone; SVZ, subventricular zone; i.p., intraperitoneal; s.c., subcutaneous; i.v., intravenous; i.c., intracerebral; i.u., intrauterine.

The African strain (MR-766) has also been shown to induce severe brain damage, including reduced brain size and cortical thinning, as well as postnatal mortality (70). However, due to extensive serial propagation in suckling mice and Vero cells, the MR766 strains used in most of the animal studies are different from the original Ugandan strain isolated in 1947, which has a mutation in a potential glycosylation site in the E protein (80, 81). The MR766 strain with the deletion of the VNDT motif within the glycosylation site exhibited reduced neuroinvasion (82).

## Brain Cells Targeted by ZIKV

### Radial Glial Cells

ZIKV has been shown to infect radial glial cells (RGCs) in both the ventricular and outer zones (55). The RGCs are primary neural progenitors, one of the earliest classes of cells to emerge from the neuroepithelium. They extend across the developing cerebral wall from the ventricular cavity to the pial surface. These cells are critical for the production and placement of neurons during brain development, and they give rise to diverse types of neuronal and glial cells (20, 83).

Examination of postmortem forebrain and SC tissues of ZIKV-infected fetus with microcephaly revealed the presence of ZIKV-NS1 and ZIKV-ENV in the neocortical RGCs. ZIKV infection of RGCs caused centrosomal depletion and mitochondrial sequestration of phospho-TANK-binding kinase 1 (TBK1), resulting in abnormal mitoses, architectural disorganization and cell death (45). Another study demonstrated that infection of RGCs by vertically transmitted ZIKV resulted in the reduction of cortical neural progenitors and subsequent defects in the brain development of the offspring mice (13). Meanwhile, ZIKV-NS2A was identified as the primary protein responsible for the reduced proliferation, premature differentiation and depletions of RGCs in the developing mouse cortex, which subsequently disrupted the positioning of newborn neurons and cortical layer organization. ZIKV-NS2A was found to exert its action by destabilizing the adherens junctions (AJs), which anchor RGCs and regulate their properties (84). AJ plays a role in regulating signaling pathways critical for neural development and its disruption has been shown to cause architectural disorganization of the developing cortex (32).

### Neural Progenitor Cells

Neural progenitor cells (NPCs), an integral population of the developing embryonic brain, have been shown to be a direct target of ZIKV. Disruption of NPC differentiation is suggested to be the major cause of microcephaly (73, 74). Different strains of ZIKV have shown the ability to infect hNPCs at varying rates and display different gene expression profiles. After 64 hours of infection, the African ZIKV strain (MR766) resulted in higher infection (69.8% at MOI of 0.02) compared to the Asian strain (FSS13025), which only caused 46.7% of infection at a higher MOI (0.04) (85). In addition, despite significant overlap in the changes in gene expression between the two ZIKV strains, the Asian ZIKV-infected hNPCs displayed less prominent changes in gene expression than the African ZIKV infected hNPCs. However, infection with the Asian strain resulted in dysregulation of an additional 10 genes involved in DNA replication and 13 genes involved in DNA repair (85). A recent study has demonstrated that the differential pattern of gene expression observed in different strains of ZIKV could be due to the differences in infection level (86). Dengue virus type 2 (DENV2) has also been shown to infect hNPCs efficiently (85, 87). However, the levels of DENV2 vRNA were lower compared to ZIKV (MR766). It was also shown that ZIKV resulted in a 30-fold increase of vRNA levels from 24 to 72 hours of infection, whereas DENV2 vRNA levels decreased over the 2 days (87). In contrast to ZIKV, which significantly downregulated genes involved in DNA replication and replication fork, DENV2 induced changes in genes related to inflammatory response and Wnt signaling (85). A study showed the ability of the Asian ZIKV strain (SZ01) to infect embryonic mouse brains, in which ~300-fold of viral RNA copies were detected 3 days post-infection and the size of the infected brains was smaller compared to the non-infected brains (74). The study revealed that ZIKV mostly targeted cells in the VZ and SVZ of the brain where the NPCs are located.

ZIKV has exhibited cell cycle arrest, apoptosis and inhibition of NPC differentiation which leads to cortical thinning and microcephaly (74). Flow cytometry analysis of ZIKV infected hNPCs demonstrated cell cycle perturbation and the results were further confirmed by gene ontology analyses which revealed enhancement of downregulated genes in cell cycle related pathways. In another study, infection of NPCs with ZIKV displayed an extended cell cycle length of about 30h compared to the control (20h), suggesting cell cycle arrest (73). ZIKV causes attenuation of hNPCs growth *via* induction of caspase-3 (73, 74, 88, 89) and ZIKV infection of hNPCs has also resulted in upregulation of genes in apoptosis-related pathways (85).

In addition, ZIKV-induced microcephaly is also associated with its ability to trigger a strong immune response in NPCs. It was found that infection of NPCs isolated from developing mouse brains with ZIKV (MEX1-44) resulted in a sharp increase of tumor necrosis factor (TNF-α) (73). TNF-α, a proinflammatory cytokine has been associated with neuronal survival and neurogenesis through activation of two distinct receptor subtypes, TNF-R1 and TNF-R2. TNF-R1 has been shown to act as a suppressor of progenitor proliferation, whereas TNF-R2 contributes to the survival of the newly formed neurons (90). In a study conducted by Kim et al., treatment of primary human NPCs with TNF-α exhibited a reduction in apoptosis by activating the NF-κB pathway (91). However, TNF-α has been shown to cause inhibition of neuronal cell number and specific neuronal cytoskeleton protein expression in the NPCs (92). In addition, TNF-α has demonstrated inhibition of neuronal differentiation of human NPCs mediated by activation of STAT3 signaling (93, 94). The overall effect of TNF-α on NPCs is most likely dependent on the cytokine levels, the affinity and relative expression of TNF-R1 and TNF-R2, as well as binding to TNF receptors and subsequent intracellular signaling (95).

ZIKV infection of NPCs caused disruption of a critical cellular quality control process called the nonsense-mediated mRNA decay (NMD) pathway. NMD is a post-transcriptional gene regulation mechanism in eukaryotes that serves as a quality-control process by destroying transcripts containing premature termination codons (PTCs) (96, 97). Notably, the NMD also plays a critical role in regulating the expression of naturally occurring transcripts (normal transcripts) (98). The disruption of the NMD pathway has been linked to microcephaly and other neurological complications. In mice, genetic ablation of NMD factors (SMG1, SMG6, UPF1, UPF2 and UPF3a), as well as exon-junction complex (EJC) components (RBM8A and MAGOH) has been shown to cause early embryonic lethality (99). NMD is essential for the early development of mice as mice embryos with mutations in NMD factors resulted in death during gastrulation or early stages of organogenesis. In mammalian brain development, mutations in NMD factors and EJC components are associated with neurodevelopment disorders. Haploinsufficiency of RBM8A and MAGOH in mice has been shown to cause microcephaly (99). As shown by Mao et al. EJC haploinsufficiency affects neurogenesis by activating the p53 dependent cell death pathway. This was evident when the loss of p53 prevented microcephaly caused by EIFA3 haploinsufficiency (100). Other than that, Doublecortin (DCX), a microtubule-associated protein that plays an essential role in neurogenesis was downregulated at both mRNA and protein levels during ZIKV infection in NPCs and fetal mouse brains (46). The downregulation of DCX in NPCs was linked to NS4A and NS5 (46). DCX downregulation was observed in NPCs infected with human cytomegalovirus (HCMV), which is a leading viral cause of birth defects and neurological dysfunction (101).

### Astrocytes

Human fetal astrocytes (HFAs) are another type of cell that can support persistent and productive infection of ZIKV for a minimum of one month and are regarded as the reservoirs for ZIKV in the fetal brain. Astrocytes are the most abundant cell type in the CNS, and they play a role in maintaining BBB functions (73, 102). They have been shown to be the first brain cell type targeted by ZIKV upon infection of newborn mice (73, 103). ZIKV infection caused an increase in the number of astrocytes in mice brains at postnatal day 3, indicating astrogliosis and brain injury (73). The HFAs were shown to be resistant to apoptosis and the interferon response, which results in chronic brain infection associated with the ZIKV neurodevelopment abnormalities. Even though flaviviruses such as West Nile West Nile virus (WNV), tick-borne encephalitis virus (TBEV) and dengue virus have been proven to induce apoptosis in human brain tissue, apoptosis induced by ZIKV in HFAs is significantly lower. This could support the prolonged virus shedding and persistence of ZIKV in the fetal brain, which can result in an increased viral load in the cortical layer as well as infection of additional cortical cells. The loss of astrocytes in the cortex can reduce the density of glial and neuronal cells, which eventually results in calcification, a common scenario seen in ZIKV caused congenital infection and microcephaly (102).

In addition, interferon response also plays an important role in ZIKV infection of the HFAs. Post-treatment with interferon did not block chronic viral infection and the cells continuously shed virus for at least a month in spite of the robust antiviral response. However, only moderate viral titers were observed and the level of ZIKV-positive cells in persistently infected HFAs was low (56). Another study demonstrated that ZIKV infection of astrocytes resulted in only limited immune cytokine and chemokine response activation (104).

### Microglial Cells

Microglial cells have been shown to be highly permissive to ZIKV infection (105, 106). These cells are the first glial cells observed in the brain and they are known as the brain’s immune cells. They are involved in the maturation of neural circuits and possess the capacity to secrete neuroactive molecules. ZIKV (H/PF/2013) infection of microglial cells resulted in the expression of high levels of interferon type I (IFN-α and IFN-β) and type II (IFN-γ), as well as neurotoxic factors with strong proinflammatory effects (TNF-α, IL-1β, IL-6, MCP-1) (105, 107). The production of IL-1β is associated with the upregulation of lysophosphatiylcholine (LPC) in microglial cells upon infection by ZIKV (105). LPC has been demonstrated to cause morphological changes in microglial cells (108) and they have also been shown to enhance neurotoxic protein aggregation (109). ZIKV infection of microglial cells also resulted in an increased production of nitric oxide (NO) and inducible nitric oxide synthase (iNOS), which is associated with the increased levels of LPC as well as proinflammatory cytokines. Induction of INOS has been shown to induce NO-mediated neuronal cell death (110).

### Glioblastoma Stem Cells

Other than the neuronal cells in the developing brain, ZIKV has also been shown to infect stem-like brain tumor cells. ZIKV efficiently infected patient-derived glioblastoma stem cells (GSCs) in a SOX2-dependent manner and induced apoptotic cell death. The study demonstrated that the knockdown of SOX2 reduced the ZIKV infectivity of GSCs, in contrast to AXL. The study found that SOX2 mediates ZIKV infection of GSCs by suppressing the innate immune response. Furthermore, ZIKV infection of glioblastoma organoids resulted in upregulation of genes in the inflammasome, TLR signaling, adaptive immune responses, and IFN responses. In addition, it was found that integrin αVβ5 mediates ZIKV internalization into GSCs. Treatment with αVβ5 blocking antibody reduced the size of glioblastoma organoids over time. These findings support the oncolytic activity of ZIKV against GSCs and that this is linked to expression of αVβ5 integrin (66). ZIKV infection of patient-derived GSCs decreased proliferation and increased apoptosis. ZIKV infection of mice with glioma, prolonged survival of the tumor-brain mice, and histopathological examination revealed ZIKV-infected tumors were significantly smaller in size (111). Overall, these findings support the idea that ZIKV infection could serve as a potential therapy for glioblastoma.

## Mechanisms of Zika Virus Neuropathogenesis

### Neuronal Apoptosis

**Figure 3:**
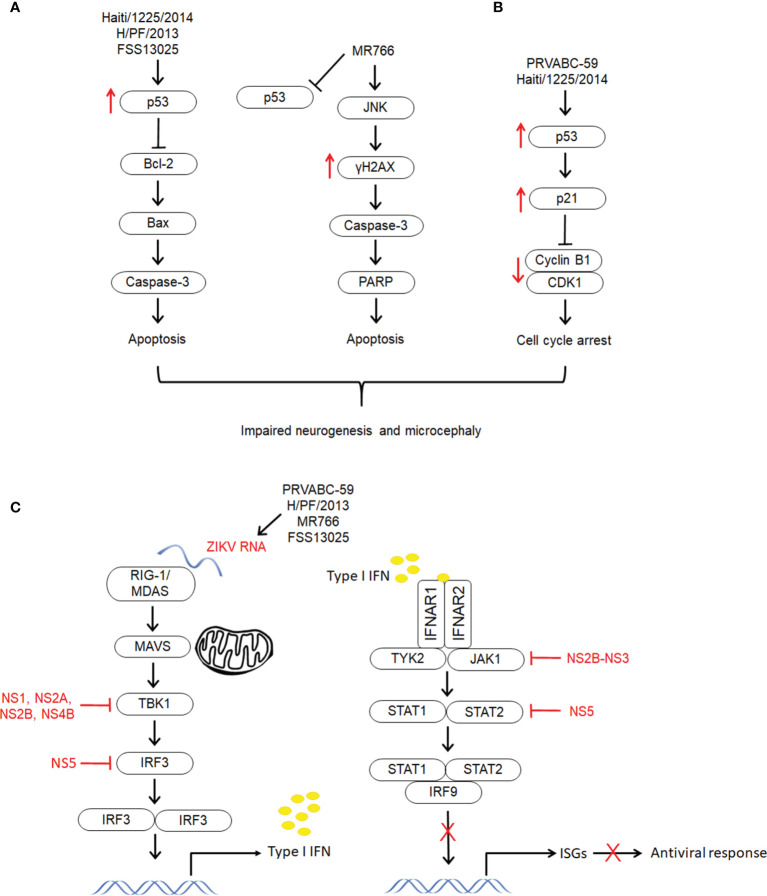
The different mechanisms exploited by different strains of ZIKV to establish infection and develop neuropathogenesis. The molecular mechanisms underlying ZIKV infection and neuropathogenesis have shown to be lineage specific. **(A)** Apoptosis. Both Asian lineage strains (HAITI/1225/2014, H/PF/2013 and FSS13025) and African lineage strain (MR766) induce apoptosis *via* activation of caspase-3. However, only the Asian strains showed upregulation of p53 and they have shown to induce intrinsic cell death pathway through regulation of Bcl-2 (114–116). On the other hand, the apoptosis induced by the African strain could be *via* JNK pathway through activation of γH2AX (115, 117, 118). **(B)** Cell cycle arrest. Unlike other Asian lineage strains PRVABC59 and HAITI/1225/2014 showed induction of cell cycle arrest through upregulation of p53 and p21Cip1/Waf1, and downregulation of cyclin B1 and cell cycle kinase CDK1 (114, 115). **(C)** Immune response. Zika virus infection has demonstrated to activate type II IFN, and supress type I and type II IFN signaling. This differential modulation of IFN signaling which is associated to destabilization of STAT2 has resulted in selective expression of ISGs and differentiated activation of immune and proinflammatory response (119). Zika virus structural proteins (NS5, NS2A, NS2B, NS4B, NS4A and NS2B-NS3) have demonstrated to modulate immune response through distinct pathways (119–121).

Zika virus (ZIKV) induces neuropathogenesis *via* various mechanisms, primarily neuronal apoptosis, cell cycle dysregulation, immune and inflammatory responses. Apoptosis has proven to be the key type of cell death in ZIKV induced developmental brain disorders, including microcephaly. In brain organoid models, ZIKV infection induced significant activation of caspase-3 and cell death, resulting in diminished cortical layers and attenuated growth (88, 112, 113). These findings were further confirmed in mouse models, in which ZIKV infection induced activation of caspase-3 and DNA fragmentation in NPCs, resulting in a decrease in the cortical NPC pool and smaller brains with damaged brain structure (13, 73, 74, 79). In the brains of pups with congenital malformations born to Brazilian ZIKV-infected pregnant mice, dysregulation of genes linked to apoptosis and autophagy was observed (72). Another study showed that cells in the intermediate zones and cortical plates (CP) of ZIKV infected embryonic mouse brains were strongly positive for caspase-3 (74). Caspase-3 expression was also increased in the parenchyma, which comprises areas of the cerebral cortex in brain tissues from fatal Zika microcephaly cases. This was accompanied by the expression of cytokines (IL-4, IL-10, IL-33, IL-37, and TNF-α) associated with apoptotic cell death (102). These findings were corroborated by Liu et al. who demonstrated the ability of ZIKV-ENV protein to induce apoptosis *via* caspase-9 and caspase-3 dependent intrinsic cell death pathway (114). In addition, ZIKV ENV protein also resulted in up-regulation of both p53 and p21^Cip1/Waf1^ and an increase in the ratio of Bax/Bcl-2 (**Figure 3A**). It has been identified that ZIKV causes mitochondrial damage and increased levels of ROS in astrocytes, resulting in DNA breaks through activation of DNA damage response (DDR) signaling leading to cell death (122).

Apoptotic cell death was evident in MR766 infection with Poly (ADP-ribose) polymerase (PARP) cleavage and caspase-3 activation (**Figure 3A**) (115). On the other hand, there were no PARP cleavage and activation of caspase-3 in PRVABC59 (the current American epidemic strain) infected hNSCs. The French Polynesia (H/PF/2013) strain of ZIKV showed activation of p53 along with an increase in caspase-3 (116). When hNPCs were exposed to Asian (FSS13025) and African (MR766) strains of ZIKV, both the strains showed signs of apoptosis, but only the Asian strain resulted in upregulation of p53 (**Figure 3A**). The role of p53 in infection of the Asian strain was further confirmed when p53 inhibitors exhibited higher potency in protecting the cells from apoptosis by inhibiting caspase-3 activation (85).

### Cell Cycle Dysregulation

The next crucial mechanism associated with ZIKV induced abnormal development of the CNS is cell cycle arrest. ZIKV-ENV protein caused G2/M arrest, which was further confirmed by downregulation of G2/mitotic-specific cyclin B1 and inhibition of phosphorylation of cell cycle kinase CDK1 (**Figure 3B**) (114). The cell cycle arrest induced by ZIKV ENV could be attributed to the upregulation of p53 and p21^Cip1/Waf1^. Increased levels of p53 phosphorylation were also observed in hNSCs infected with PRVABC59. However, infection with MR766 demonstrated reduced p53 phosphorylation. Following increased phosphorylation of p53, upregulation of p21 and PUMA was observed in PRVABC59 infected hNSCs suggesting, p53-mediated cell cycle arrest. Infection of hNPCs with PRVABC59 exhibited expression of cell cycle associated genes, including CDKN1B, CDKN2B, GADD45A and WEE1 (115). Another study revealed that 11 downregulated genes in tissues from ZIKV-linked microcephaly are involved in cell cycle regulation (74, 88). In addition, it has been proven that the growth attenuation of hNPCs is partly due to cell cycle dysregulation (88). NPC cell cycle arrest was observed in the mice embryonic brains, which displayed microcephalic phenotypes following ZIKV infection. In these mice, fewer mitotic cells in the VZa, reduction in the number of NPCs in the M phase and suppression of NPC proliferation were observed (74).

Strong evidence supports that primary microcephaly is linked to cell cycle dysregulation. Many of the primary microcephaly genes encode proteins involved in cell cycle regulation, centriole biogenesis and mitosis (123). Centrosomal abnormalities can lead to impairment of mitosis, which is a hallmark of autosomal primary recessive microcephaly (MCPH). In addition, a significant proportion of microcephaly genes are ontologically linked to the centrosome (124). In a vertically transmitted ZIKV infection case with microcephaly and other congenital abnormalities, a mutation in cyclin-dependent kinase 5 regulatory subunit-associated protein 2 (CDK5RAP2) was detected (44). CDK5RAP2 is a centrosomal protein that plays a critical role in the cell cycle through the regulation of microtubule function. Mutations in CDK5RAP2 have been shown to reduce the number of NPCs and have been linked to primary microcephaly (42, 43, 125, 126). ZIKV-NS3 was found to interact with centrosome regulatory proteins such as CEP192, a major regulator of centrosome biogenesis and spindle organization (33); OFD1, a centriolar satellite protein and regulator of centriole architecture (127); and CEP85, a regulator of centriole duplication (35, 128). To further support these findings, Golubeva et al. identified the interaction of ZIKV proteins with proteins that are associated with mitosis as well as primary microcephaly (129).

### Immune Response and Signaling Pathways

An intense immune response to cell injury was observed in the postmortem brains of neonates with CZS, as demonstrated by the gliosis and inflammatory infiltrate in the meninges, cerebral hemispheres, and spinal cord (1). Modulation of the host immune response by ZIKV has proven to be the key mechanism for ZIKV neuropathogenesis. Global transcriptome analyses of RNAs isolated from ZIKV infected developing brains demonstrated a large number of differentially expressed genes associated with the immune response (73, 74). The top 10 most significantly upregulated genes (OASl2, USP18, IFIT1, MX2, OAS1b, IFIT3, LIGP1, DDX60, IFI44, IRF7) are linked to interferon response (73). In addition, genes that are involved in cytokine productions such as *IL1B*, *TNF*, *CXCL10*, *IFNB1* and *TLR3* were also upregulated (74). Dang et al. discovered that ZIKV induced activation of an innate immune receptor, TLR3 in mouse neurospheres and human organoids, which resulted in the disruption of 41 genes associated with neurodevelopment and, as a result, a reduction in organoid volume. Treatment with a TLR3 competitive inhibitor attenuated the shrinkage of ZIKV-infected organoids (130). These findings suggest the potential role of TLR3 in ZIKV neuropathogenesis.

ZIKV (PRVABC-59) NS5 strongly inhibited IFN-β signaling and activated IFN-γ signaling (119). It also caused suppression of IFN-λ without having any effect on TNF-α-induced activation of NF-κB activity. The differential modulation of type I and type II IFN signaling is attributed to the ability of NS5 to destabilize STAT2 but not STAT1. The destabilization of STAT2 affected the formation of both STAT1-STAT2-IRF9 and STAT1-STAT1 complexes, which may control the relative activities of ISRE and GAS in different ISGs (**Figure 3C**) (119). STAT2 protein levels were also reduced by NS5 from other strains of ZIKV (H/PF/2013 and MR766) (41).

In addition to NS5, other ZIKV nonstructural proteins (NS2A, NS2B, NS4B and NS4A) from Cambodian FSS13025 have also been shown to suppress the production of IFN-β in HEK-293T cells by targeting distinct cellular components from the retinoic acid‐inducible gene 1 receptor (RIG-1) pathway (**Figure 3C**). It was also discovered that the PRVABC-59 and Dakar 41525 strains suppressed IFN-β induction by binding to TBK1 in HEK-293T cells (120). Another study demonstrated that NS1 and NS4B of Z1106033 inhibited retinoic acid-inducible gene 1-like receptors (RLR)-induced production of IFNβ by interacting with TBK1 and blocking its oligomerization. It was also shown that ZIKV MR766 infection reduced phosphorylation of Janus kinase 1 (JAK1) and STAT1 in A549 cells. These observations are associated with NS2B-NS3 (Z11060330), which caused a reduction in the protein levels of JAK1 and subsequently inhibited phosphorylation of JAK1 and STAT1 (**Figure 3C**). In addition, NS2B-NS3 also reduced the expression of ISG15, IFIT1, IFIT2 and Viperin (121). A study demonstrated that infection of human DCs with PRVABC59, P6-740, MR-766, and Dakar 41524 strains resulted in the induction of notable IFNB1 gene transcription but caused inhibition of type I IFN protein translation (131). On the other hand, MR766, PRVABC59 and R103451 strains in astrocytes and MEX1-44 in NPCs led to a significant increase in the secretion of IFN-β levels (73, 117). These findings indicate that secretion of IFN-β seems to be dependent on the strain and cell type.

As the innate immune response is compromised during ZIKV infection, the adaptive immune response has an important role in controlling the infection. Suppression of type I IFN response in Rag1−/− mice, which lack both T cell and B cell responses, resulted in weight loss and increased levels of viral RNA in spleen, lymph node, brain and testes. In these mice, neurons were the main target for ZIKV, and the astrocytes and microglia showed signs of activation, suggesting CNS damage (132). Notably, it has been shown that pregnancy-linked immunotolerance impacts the T cell response to ZIKV infection in the uterus (133). Furthermore a, reduction of CD4+ and CD8+ T activation and proliferation were observed in pregnant mice compared to non-pregnant controls (132). The influence of pregnancy on cell-mediated immunity could increase the virus spread to the fetus leading to adverse pregnancy outcomes.

### Neuroinflammation

In recent studies, it has been found that ZIKV induced inflammatory responses may be responsible for the disruption of the BBB. ZIKV has been shown to efficiently infect BBB cells, including endothelial cells, pericytes and astrocytes, leading to upregulation of inflammatory cytokines (IL-6 and IL-8) and chemokines (CCL5 and CXCL10) both *in vitro and in vivo* (134). These inflammatory molecules modulate the integrity of the BBB and act as immune cell recruitment. In addition, ZIKV infection also resulted in upregulation of cell adhesion molecules (CAMs), which are involved in leukocyte docking to the BBB and contribute to immune cell CNS infiltration and neuroinflammation (134). Examination of postmortem brain samples of ZIKV-infected neonates with CZS revealed a decrease in expression of genes related to ECM organization and collagen formation, such as collagen encoding genes, which are important for the development of the brain and the BBB. A significant increase in PTPRZ1, which is involved in the modulation of inflammation in the CNS was observed (1). The African strain of ZIKV demonstrated greater upregulation of certain inflammatory and adhesion molecules than the Asian strain (134).

In a study by Gurung et al. fetuses born to ZIKV-infected pregnant olive baboons displayed neurological damage, including defects in radial glia, disorganised neuron migration to cortical layers, and pathology in immature oligodendrocytes. Indicators of severe neuroinflammation including astrogliosis, increased microglia and IL6 were observed in the fetuses (135). Similarly, in mice, intrauterine ZIKV infection during pregnancy resulted in neuroinflammation and cortical thinning in postnatal brains (78). Importantly, neurodevelopmental defects in fetuses were observed despite the absence of detectable ZIKV RNA, suggesting the possible role of neuroinflammation in causing long-term sequelae (78, 135).

An inflammatory form of programmed cell death, pyroptosis has been shown to play a role in ZIKV associated developmental disorders and microcephaly. Brain tissue specimens from ZIKV-infected mice had significantly elevated inflammasome-associated genes, including IL1B, IL-18, CASP1, ASC, and GSDMD. Additionally, cleavage of caspase-1 and GSDMD occurred in the ZIKV-infected brains. Other than that, caspase-1 was intensely stained in ZIKV-infected neutrospheres. Furthermore, LDH release was observed in ZIKV-infected neurospheres (136). These findings support the occurrence of pyroptosis cell death during ZIKV infection. In caspase-1-deficient mice, severe brain atrophy was significantly reduced following ZIKV-infection compared with ZIKV-infected wild-type mice. Histopathological examination revealed that Caspase-1 deficient mice did not exhibit any inflammation-induced damage. Furthermore, treatment of ZIKV-infected mice with a selective caspase-1 inhibitor (VX-765) reduced ZIKV-induced severe brain atrophy and reversed neuroinflammation (136).

### Endoplasmic Reticulum Stress and Activation of the Unfolded Protein Response

The other mechanism is linked to the remodeling of the endoplasmic reticulum (ER) structure by ZIKV for its replication. This, in turn, causes the accumulation of misfolded virus polyproteins in the ER lumen, resulting in ER stress and activation of the unfolded protein response (UPR). Elevation of the expression of ER stress markers such as GRP78, calreticulin, calnexin and protein disulfide isomerase (PDI) was observed in ZIKV-infected neural progenitors. This series of events subsequently leads to the disruption of neurogenesis. Other than UPR, ER stress-induced during ZIKV infection also leads to other cellular processes, including the formation of stress granules and reticulophagy to repair stress-induced damage and restore normal cellular functions. However, ZIKV proteins subvert these processes to allow continuous viral replication and protect the virus from host cell innate defense mechanisms. Prolonged ER stress may eventually result in paraptosis-like death (137).

### Modulation of Mitochondrial Dynamics

Several studies have shown ZIKV-induced disruption of mitochondrial dynamics in different cells, including human retinal pigment epithelial (RPE) cells, human iPSC-derived astrocytes (122). human neural stem cells (NSCs) and human glioblastoma cells (SNB-19) (49). ZIKV-induced ER stress causes calcium (Ca2+) release, which can be taken up by mitochondria, resulting in an increase in ROS production and mitochondrial-dependent cell death. Furthermore, ZIKV replication requires energy, which leads to ATP synthesis by OxPhos and increased oxygen flux. However, when the mitochondrial reserve capacity decreases, it leads to mitochondiral failure (122). Another study found that ZIKV disrupts mitochondrial dynamics by decreasing the levels of mitofusin-2 (MFN2) proteins (49). MFN2 plays an important role in maintaining the integrity of mitochondrial morphology and function by mediating mitochondria fusion. MFN2 is highly expressed in the brain (47) and it has been shown to be essential in embryonic development (48), neuronal maturation and synapse formation (138). Its deficiency has been shown to cause an increase in the levels of ROS and mitochondrial dysfunction and has resulted in a range of congenital eye defects (139), disruption of placental development and is associated with spontaneous abortion (140). A study demonstrated that Mdivi-1, a small molecule that inhibits mitochondrial fission blocked mitochondrial fragmentation and reduced ZIKV induced cell death (49). This finding suggests that maintenance of normal mitochondrial dynamics could offer a potential therapeutic strategy for ZIKV infection. Chatel-Chaix et al. demonstrated that DENV and ZIKV induced similar mitochondrial elongation. In this study, it was found that DENV-NS4B was responsible for inducing mitochondrial elongation through inactivation of the mitochondrial fission factor Dynamin-Related Protein-1 (DRP1) (141). The study further demonstrated that DENV-induced mitochondrial elongation enhanced DENV replication and reduced RIG-1 dependent activation of interferon responses. ZIKV may also use modulation of mitochondrial morphodynamics to disrupt innate immunity (141).

## The Mechanisms of Zika Virus-Associated-Guillain-Barre Syndrome in Adults

Other than neurological complications in new-borns and adverse pregnancy outcomes, ZIKV has also been associated with Guillain-Barre syndrome (GBS) in adults. ZIKV outbreaks in French Polynesia and Latin America witnessed an increase in the incidence of GBS (142, 143). Epidemiological studies reported an increase between 2.0- and 9.8-fold in the ZIKV-associated GBS in 7 countries in the Americas (144).

Analysis of plasma samples from Zika patients with GBS showed the presence of higher levels of anti-ganglioside IgM/IgG antibodies compared with Zika patients without GBS (145). Similarly, another study found a several-fold increase in the levels of IgG autoantibodies to brain gangliosides in serum of the Zika patients (146). Another study found anti-glycolipid antibody activity against, particularly GA1 (142). An in silico analysis found that the glycan loop (GL) region of the E protein contains an IVNDT motif which is conserved in human neuronal proteins, namely Heat Shock 70 kDa protein 12A (HSP70 12A) and voltage-dependent L-type calcium channel subunit alpha-1C (Cav1.2) (147). A study by Lucchese and Kandu found a significant peptide overlap between ZIKV and human proteins that when altered are linked to GBS (148). These findings suggest the possible role of molecular mimicry as one of the potential mechanisms of ZIKV-associated GBS.

Antibody-dependent enhancement (ADE) of Zika has been proposed as another possible mechanism for ZIKV-associated GBS. ADE occurs as a results of circulating antibodies from previous immunological responses binding to the virus, but it is not capable of neutralizing the infection. In this context, higher titers of neutralizing antibodies to both ZIKV and DENV2 were detected in ZIKV patients with GBS compared to non-GBS ZIKV patients (149). Another study by Anaya et al. found ZIKV patients with GBS had IgG antibodies against both DENV and ZIKV. Interestingly, the study also found the presence of IgG antibodies against M. pneumonia, indicating M. pneumoniae exposure as a high risk for developing GBS following ZIKV infection. Although a study by Meyer Sauteur supports the link between M. pneumoniae infection and GBS (150), more investigations are required to understand its role in ZIKV-associated GBS.

## Conclusion

Zika virus (ZIKV) has evolved to induce new clinical syndromes, particularly in newborns. Accumulating evidence support that ZIKV interacts with key host proteins to induce neuropathogenesis through various molecular mechanisms, including neuronal apoptosis, cell cycle dysregulation, exploitation of host immune response and activation of inflammatory response. These mechanisms were shown to be dependent on the types of cells, strains and infection rate. It is also evident that the ZIKV-induced anomalies are also the result of indirect effects of modulation of host immune response and inflammatory process, rather than just the virus itself. These findings suggest that a combination of different mechanisms may be responsible for the neuropathogenesis of ZIKV. However, more in-depth studies are required to fully understand the distinct molecular pathways involved in ZIKV induced infection in different brain cells and to further validate the differences observed in different strains of the virus.

In addition to brain proteins, it is also crucial to identify other host factors that drive inflammation and immune response during ZIKV infection. Identification of host proteins is important for developing effective host-directed antivirals and for drug repurposing for the treatment of ZIKV infection, particularly to prevent neurological complications in newborns and of the possible long-term effects. In addition, the combination of the host-factors-targeting agents with drugs that directly target viral enzymes could lead to a more effective therapeutic regimen to fight ZIKV as well as other flaviviruses. Importantly, given the ability of ZIKV to alter genes in the brain cells associated with CNS development, it is crucial for long-term neurodevelopmental follow-up of ZIKV-exposed infants. Notably, the absence of microcephaly at birth with prenatal exposure to ZIKV does not preclude the presence of ZIKV-associated brain abnormalities. Hence, it is crucial for long-term neurodevelopmental follow-up of ZIKV-exposed infants.

## Author Contributions

Conceptualization by TK and VB. Methodology by TK. Writing - original draft preparation by TK. Writing, review, and editing by TK, NA, WJ, and VB. Supervision by VB and WJ. All authors contributed to the article and approved the submitted version.

## Conflict of Interest

The authors declare that the research was conducted in the absence of any commercial or financial relationships that could be construed as a potential conflict of interest.

## Publisher’s Note

All claims expressed in this article are solely those of the authors and do not necessarily represent those of their affiliated organizations, or those of the publisher, the editors and the reviewers. Any product that may be evaluated in this article, or claim that may be made by its manufacturer, is not guaranteed or endorsed by the publisher.
